# Hands train the brain—what is the role of hand tremor and anxiety in undergraduate microsurgical skills?

**DOI:** 10.1007/s00701-018-3609-6

**Published:** 2018-07-02

**Authors:** John Hanrahan, Michail Sideris, Terouz Pasha, Parmenion P. Tsitsopoulos, Iakovos Theodoulou, Marios Nicolaides, Efstratia-Maria Georgopoulou, Dimitris Kombogiorgas, Alexios Bimpis, Apostolos Papalois

**Affiliations:** 1 Faculty of Life Sciences and Medicine, King’s College London, Strand, London, WC2R 2LS UK; 2 Women’s Health Research Unit, Queen Mary University of London, Mile End Rd, London, E1 4NS UK; 3 Section of Neurosurgery, Department of Neuroscience, Uppsala University, 752 36 Uppsala, Sweden; 4 Barts and The London School of Medicine and Dentistry, Queen Mary University of London, Mile End Rd, London, E1 4NS UK; 5 Medical School, National and Kapodistrian University of Athens, 157 72 Athens, Greece; 6 Department of Neurosurgery, Metropolitan Hospital, 18547 Athens, Greece; 7 Department of Neurosurgery, General Hospital of Tripoli, Erythrou Stavrou str., 22100 Tripoli, Greece; 8 Experimental Research Centre ELPEN, Athens, Greece

**Keywords:** Surgical education, Tremor, ESMSC, Microsurgery, Anxiety, Dexterity

## Abstract

**Introduction:**

Physiological hand tremor occurs naturally, due to oscillations of the upper extremities. Tremor can be exacerbated by stress and anxiety, interfering with fine motor tasks and potentially impact on surgical performance, particularly in microsurgery. We investigated the link between tremor, anxiety and performance in a neurosurgical module as part of an international surgical course.

**Methods:**

Essential Skills in the Management of Surgical Cases (ESMSC) course recruits medical students from European Union (EU) medical schools. Students are asked to suture the dura mater in an ex vivo swine model, of which the first suture completed was assessed. Questionnaires were distributed before and after the module, eliciting tremor risk factors, self-perception of tremor and anxiety. Johnson O’Connor dexterity pad was used to objectively measure dexterity. Direct Observation of Procedural Skills (DOPS) was used to assess skills-based performance. Anxiety was assessed using the Westside Test Anxiety Scale (WTAS). Tremor was evaluated by four qualified neurosurgeons.

**Results:**

Forty delegates participated in the study. Overall performance decreased with greater subjective perception of anxiety (*p* = 0.032, rho = − 0.392). Although increasing scores for tremor at rest and overall WTAS score were associated with decreased performance, this was not statistically significant (*p* > 0.05). Tremor at rest did not affect dexterity (*p* = 0.876, rho = − 0.027).

**Conclusions:**

Physiological tremor did not affect student performance and microsurgical dexterity in a simulation-based environment. Self-perception of anxiety affected performance in this module, suggesting that more confident students perform better in a simulated neurosurgical setting.

## Introduction

Multiple factors influence medical students when deciding upon a career pathway in surgery. Prospective applicants must consider the physical demands the career requires, particularly when considering specialities that demand fine motor skills to perform complex tasks, such as in neurosurgery. Physiological tremor occurs naturally in humans, which can be exacerbated in times of stress [10]. Whilst several hypotheses exist discussing potential mechanisms responsible for hand tremor [1, 5, 16, 18], the exact cause is unclear. Although physiological tremor does not usually interfere with day-to-day motor function, it can impact surgical performance, particularly when fine motor skills are required for delicate tasks [30].

Technology plays an important adjunct to neurosurgery, with the use of microscopes and endoscopes commonplace in modern practice. Microsurgery, a core component of neurosurgery, involves manipulation of instruments and surgical sites through fine movements under a high-powered microscope. Hand tremor has been described as the ‘enemy of microsurgery’, owing to exacerbation of tremor under the microscope [20]. Tremor has also been shown to amplify during minimally invasive surgery due to moments magnified by long instruments [30]. Thus, tremor may have an important impact on the neurosurgeon’s daily practice.

Methods of reducing intraoperative tremor have been developed through the elucidation of factors which exacerbate physiological tremor [8–10]. Anxiety has been described as a risk factor for physiological tremor, with pharmacological treatment suggested to provide some benefit to neurosurgical trainees prone to anxiety [10]. Yet, in simulated educational environments, anxiety has been shown not to affect undergraduate student performance [6]. Surgical tremor has been shown previously to reduce with confidence [30], suggesting familiarity of the procedure and equipment potentially playing a role in the physical ability to carry out surgical tasks.

Whilst studies have evaluated tremor in surgical residents and trainees [8, 10, 30], the importance of tremor in undergraduate medical students on their ability to perform microsurgical tasks remains unclear. Essential Skills in the Management of Surgical Cases (ESMSC) [29] is an international surgical course which aims to provide a holistic surgical education to undergraduate medical students.

This study aims to investigate the relationship between physiological hand tremor of undergraduate medical students with performance of a microsurgical suturing task during the ESMSC course. Additionally, we compared objective and subjective assessment of anxiety to the degree of physiological hand tremor.

## Methods

### Course concept

ESMSC is a biannual course aiming to provide holistic surgical education to undergraduate medical students, which runs at the Experimental Research Centre ELPEN biannually, as a part of the Network for Accredited Skills Centres in Europe (NASCE). It combines high and low fidelity in vivo, ex vivo and dry lab simulation modules with applied surgical science and basic knowledge interactive workshops. The current curriculum (cores integrated for research—Ci4R) is set up in an ergonomic way to combine 40 learning modules which promote multidisciplinary learning strategies and facilitate various research projects as part of the course [6, 24, 26–28]. This concept has been previously described [27], and it has received accreditation by the European Council for Continuous Medical Education (EACCME). The faculty is invited based on recommendation letters and selection is performed by the course committee to meet the standards and the needs of each series.

### Student selection

Students from European Union (EU) Medical Schools are invited to apply online (www.esmsc.gr). Selection involves a competitive process which requires applicants to submit a curriculum vitae (CV) and personal statement.

All students attending the seventh cycle of ESMSC were invited to take part in the study. Application of ethical approval met directive 63/2010, PD 56/April 2013 declaration, according to local policy. The latest license reference number is 485715/9/2017, MS, AP et al.

### Questionnaire design

Structured questionnaires were designed based on previous published papers and distributed before and after the workshop. These questionnaires were eliciting risk factors for tremor [10], self-perception of anxiety [6] and hand tremor.

### Module design

**Fig. 1:**
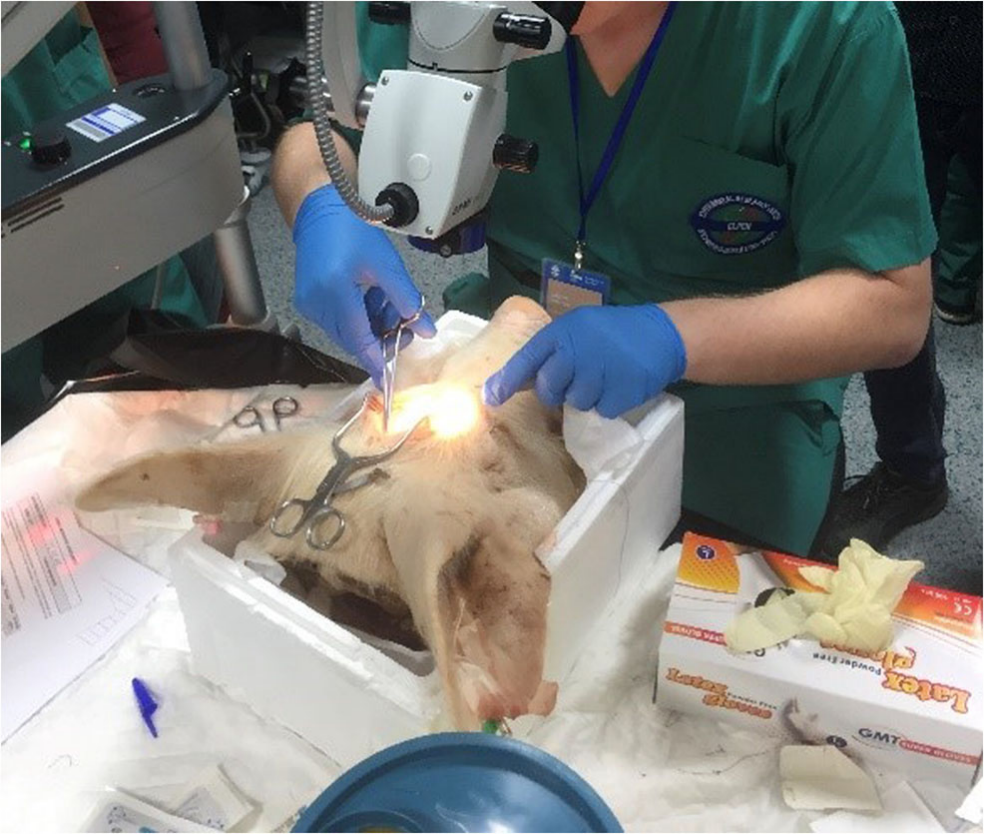
Suturing of the dura mater under the microscope

The module required students to suture the dura mater of an ex vivo swine model under the surgical microscope (Fig. 1). To ensure we received a homogeneous sample with regard to previous experience, all students had been exposed to equal time-for-practice prior to the module. Each student had used the microscope previously on a lower fidelity suturing model (introduction to microsurgery module), alongside basic suturing teaching (basic suturing module) before the assessment. Students were instructed to suture at least one complete knot under the microscope which was assessed on an individual basis by a consultant neurosurgeon. Performance was assessed using the Direct Observation of Procedural Skills (DOPS) tool [2, 13, 23, 25, 31], which is part of the Intercollegiate Surgical Curriculum Program (ISCP). This is a validated tool which forms a core aspect of postgraduate workplace-based assessment in the UK.

### Physiological tremor assessment

Students’ tremor was evaluated using a Likert scale. Although this was not validated prior, assessors reached consensus on using the scale prior to the module, and to ensure homogeneity, each mark was discussed in a 5-min debrief session upon completion of the module. Students were blinded to the assessor’s score until after the workshop. Tremor was assessed (i) at rest, (ii) when holding the surgical instruments and (iii) on the first throw of the suture.

### Anxiety assessment

Anxiety was assessed using the Westside Test Anxiety Scale [7], a validated tool which students answer 10 questions structured using a Likert-scale. This was distributed to all students prior to the workshop. Students also reported their perception of anxiety before and during the workshop through structured questionnaires which were designed for this study.

### Dexterity assessment

Dexterity was assessed using the Johnson-O’Connor Tweezer test which has been used previously as part of prior research initiatives run alongside ESMSC, with the methodology outlined [6]. This test involves students transferring pins from one 10-by-10 pad to another in 5 min using forceps. Students underwent the task twice, with the second attempt taken as their dexterity score.

### Statistical analysis

Statistical analysis was performed on IBM SPSS for Macintosh v.2 (IBM corp., Armonk, NY, USA). Simple univariate descriptive statistics were used to assess data. Correlations were explored with bivariate Spearman’s associations.

## Results

Forty delegates (female = 14, male = 26) participated in the study. Five were left-handed and 35 right-handed. Mean height of delegates was 174.2 ± 9.79 cm and mean body mass 71.7 ± 17.3 kg. Seven were in their third year of medical school (first clinical year), 29 in their fourth year and four in their fifth year.

Sixteen (48.5%) students reported caffeine consumption in the hour before the workshop. However, this did not affect overall performance, tremor at rest or WTAS score (*p* > 0.05). Sleep deprivation was the only risk factor reported by the cohort that could potentially impact performance, but upon analysis, this did not affect any performance outcome (*p* > 0.05). Table 1 summarizes responses related to potential risk factors as well as subjective perception for tremor and anxiety, as stated by delegates prior to the module.

**Table 1:** Risk factors for tremor before workshop (questionnaire responses) and tremor/anxiety self-assessment

	I have consumed alcohol in the last 24 h	I have consumed caffeine in the last hour	I feel deprived of sleep	I feel anxious	I feel hungry	I feel thirsty	I feel stressed	I felt anxious during the workshop	I feel I have shaky hands
Median (IQR)	–	–	4 (3–4.5)	2 (1–3)	3 (2–4)	3 (3–4)	3 (2–3)	3 (2–3.5)	2 (2–4)
Freq. yes	8	16	–	–	–	–	–	–	–
Freq. no	26	17	–	–	–	–	–	–	–

Assessment of tremor at rest, tremor when holding surgical instruments and tremor when throwing the first suture was in agreement (*P* < 0.001, Spearman’s rho ~ 1). Therefore, tremor at rest was used for descriptive and bivariate analysis. Table 2 summarizes the objective assessments of tremor, dexterity, anxiety and performance.

**Table 2:** Anxiety, tremor, performance and dexterity assessment

	Performance measures	
	Median score	Interquartile range
Overall Westside Test Anxiety Score (WTAS)	2.2/10	1.9–2.7
Tremor at rest	2/5	1–2
DOPS overall performance	2	2–3
Time taken (minutes)	5	4–6
Johnson O’Connor Dexterity Score	75/100	66–82.5

There was no statistically significant association between the subjective and objective assessment of tremor, although results keep in agreement (*p* > 0.05, rho = 0.189–0.146). Despite the overall agreement between WTAS scores and delegates’ subjective perception of anxiety, it did not reach statistical significance (*p* > 0.05, Spearman’s coefficient = 0.141 and 0.048). Overall performance was decreased in a statistically significant manner against delegate subjective anxiety perception (*p* = .032, rho = − 0.392). Although increasing scores for tremor at rest and overall WTAS anxiety were associated with slightly decreased performance, those results did not reach statistical significance (*p* > 0.05). Tremor at rest did not affect dexterity (*P* = 0.876, rho = − 0.027). These associations are summarized in Table 3.

**Table 3:** Bivariate comparison of performance measures (* = *p* < 0.01)

Correlation coefficient (*p* value)	DOPS overall performance	Time taken (minutes)	Overall Westside Test Anxiety Score (WTAS)	Tremor at rest	I feel I have shaky hands	I was feeling anxious	Johnson O’Connor Dexterity Score
DOPS overall performance	–	0.062 (0.750)	− 0.155 (0.351)	− 0.045 (0.796)	− 0.203 (0.273)	− 0.501 (0.003)*	− 0.091 (0.596)
Time taken (minutes)	0.062 (0.750)	–	− 0.325 (0.086)	0.080 (0.691)	− 0.081 (0.706)	− 0.280 (0.185)	0.011 (0.957)
Overall Westside Test Anxiety Score (WTAS)	− 0.155 (0.351)	− 0.325 (0.086)	–	− 0.024 (0.890)	0.048 (0.790)	0.275 (0.121)	− 0.19 (0.911)
Tremor at rest	− 0.045 (0.796)	0.080 (0.691)	− 0.24 (0.890)	–	0.089 (0.635)	0.280 (0.115)	− 0.27 (0.876)
I feel I have shaky hands	− 0.203 (0.273)	− 0.081 (0.706)	0.048 (0.790)	0.089 (0.635)	–	0.183 (0.360)	0.087 (0.643)
I was feeling anxious	− 0.501 (0.003)*	− 0.280 (0.185)	0.275 (0.121)	0.280 (0.115)	0.183 (0.360)	–	− 0.138 (0.451)
Johnson O’Connor Dexterity Score	− 0.091 (0.596)	0.011 (0.957)	− 0.019 (0.911)	− 0.027 (0.876)	0.087 (0.643)	− 0.138 (0.451)	–

## Discussion

Hand tremor can impact on fine motor skills in surgery, particularly in procedures involving magnification of small surgical sites and use of instruments that can amplify tremor. This is particularly true for microsurgery, an integral part of neurosurgery, where hand tremor may compromise stability of instruments and likely the quality and efficacy of the procedure [8, 22].

Tremor assessment in our cohort revealed that students commonly had minimal tremor during the workshop. Whilst our assessment tool of tremor was not previously validated, assessors were in consensus in using the tool, and the objective and self-perception of tremor were in agreement. Both objective and self-perception of hand tremor did not impact overall performance during the workshop, time taken to complete the workshop or dexterity score. This finding is important for undergraduate medical students, as some students may be deterred from entering neurosurgery because they may feel they do not have steady enough hands. It should be mentioned though that our findings are limited to the undergraduate level, and further studies should examine this association in a higher fidelity setting, with incorporation of more experienced doctors performing microsurgery for comparison.

Risk factors for the enhancement of tremor during surgery have been investigated previously, with the findings of this systematic review forming the basis of our risk factor assessment for tremor [10]. Although the majority of risk factors did not affect our cohort’s performance, students frequently agreed with the statement ‘I feel deprived of sleep’. Whilst this may have been due to the intensity of the 3-day course students had enrolled on, this factor did not impact performance (*p* > 0.05). Micko et al. recently examined the impact of sleep interruption on microsurgical performance in a neurosurgical simulator. Improved performance was observed in students and trainees with sleep interruption, compared to their scores when well-rested [21], supporting the lack of impact sleep deprivation had on performance seen in this study.

Verelli et al. demonstrated reduction in tremor intensity in all but one subject on their second attempt and this was postulated to be a result of reduced anxiety [30]. Anxiety can exacerbate tremor, with pharmacological intervention previously suggested as a method to reduce tremor in trainee surgeons [8]. Our study found that self-perception of anxiety at the time of the workshop correlated with student performance. A similar trend was seen when comparing the WTAS score with performance, but this was not statistically significant. Whilst anxiety played a role in the performance of the students in our study, it is important to dissect performance anxiety from an anxious personality. Although the WTAS provides an objective and validated assessment of student anxiety, this score aims to identify students with anxiety impairments drawing on wider aspects of their behaviour [7].

Self-perception at the time of workshop is a more precise measure of how the student felt at the time of assessment. The association between self-perception of anxiety and performance may represent student confidence, and suggests student anxiety may hold the student back in the summative or performance setting. Confidence is influenced by multiple factors, one of which is prior experience to the skill being tested. Lee et al. evaluated the impact of dexterity on the career interests of medical students. Perceived personal skill set influenced career choice, where those interested in surgery had a greater perceived innate manual dexterity. However, there was no difference in objective innate manual dexterity between those interested and those not interested in a career in surgery, suggesting future surgeons perception of their skillset was a key determining factor in career path selection in the study [17]. Similar to the association in this study between self-perceived anxiety and performance, this may represent student confidence. Yet, these results must be interpreted with caution, to avoid overestimation of ability and mitigate dangerous behaviour in future neurosurgeons.

Surgical careers require physical abilities and stamina to perform operations safely, effectively and in a timely manner. It is important for both prospective neurosurgeons and training program selection panels to understand the qualities required for long-term success in a surgical career. Thus, investigation into dexterity is an area of interest in medical education research [6, 10, 14, 17, 19, 32]. Assessment of dexterity has been shown to be feasible during surgical residency interviews, providing valuable information [15]. The importance of this was highlighted by Buckley et al. where they concluded that applicants with lower baseline scores in dexterity assessments required a longer period of training time, some even unable to reach proficiency even after repeated attempts [4]. There is value in identifying qualities which support pursuing a career in surgery, alongside elucidating predictors of incompatibility with a surgical career.

Whilst our cohort was naïve to the microscope, with identical baseline education on using the microscope in a previous workshop and suturing training during the ESMSC course, the pre-course abilities of students may vary due to the different surgical education and exposure experienced nationally and internationally, alongside innate dexterity. However, no association between dexterity score and all performance outcomes was observed, adding to the controversial literature on the predictive value of dexterity assessments. Medical student dexterity has been suggested to predict surgical skills despite past experiences in some studies [14, 19, 32], whilst others report no impact on surgical skills’ performance [32]. Our findings indicating innate characteristics of the student (dexterity and hand tremor) did not impact on any performance outcome suggest practical skills and success in neurosurgery are attributed to other, potentially modifiable, factors, such as anxiety. However, further studies are needed to evaluate this association in a simulated setting of a higher fidelity. Moreover, our assessment of microsurgical dexterity was limited by the fact that students were required to complete only a single knot under the microscope even if in some occasions more knots were completed. However, at the undergraduate setting, we believe this measure suffices assessing dexterity and microsurgical skills developed from the previous workshops (basic suturing module and introduction to microsurgery module), as initial use of the microscope and microsurgical suturing is a difficult task for the student naïve to the microscope, with a median time taken of 5 min (Table 2) to complete the task. Additionally, conducting the study within the time constraints of the ESMSC course limited the time available to assess individuals. Future studies should incorporate more detailed assessment of microsurgical skills.

Elucidating factors affecting microsurgical performance through primary data collection as in our study or evidence synthesis permits optimization of the surgeon’s behaviour and environment. Belykh et al. outlined factors affecting microsurgical performance and provided recommendations to improve performance [3]. They highlighted the negative impacts of alcohol on physiological tremor, the lack of evidence of the benefit of drugs such as beta-blockers and other factors influencing performance. We feel such findings encourage positive behaviours, with strategies such as active confidence building and optimization of the surgical environment as alternatives to medication and alcohol consumption.

For prospective neurosurgeons, the present study highlighted that self-perceived anxiety associated with overall performance in a microsurgical suturing simulation, and that physiological tremor had no impact on performance or dexterity. Students should seek out methods to minimize feelings of anxiety for practical skills, which can be applied to their surgical speciality interview and future operating. It is clear medical educators must find ways to build student confidence whilst developing their competence in surgical skills without fostering arrogance. Moreover, educators should ensure development of student confidence mitigates anxiety and encourages engagement, but maintaining mindfulness to avoid overestimation of abilities. Formal surgical skills courses can bridge the transition from medical school to a surgical career, and inspire confidence [11, 12]. The stepped-approach to microsurgery provided through the ESMSC gave students a foundation in suturing and using the microscope before combining the two skills into the microsurgical suturing workshop.

## Conclusion

The current study demonstrated that in a neurosurgical simulation, objective assessment and self-perception of hand tremor did not influence student dexterity or overall performance in microsurgery. This finding is important for potential neurosurgeons, particularly students who feel they do not have steady enough hands for neurosurgery. Self-perceived anxiety was associated with overall performance, suggesting student confidence is important in simulation-based performance. Further research should investigate these associations in a higher fidelity setting evaluating more experienced physicians. Additionally, surgical education initiatives should focus on inspiring confidence in practical skills for students at an early stage to foster positive attitudes towards surgical specialities and engaging in practical skills.

## Abbreviations

*Ci4r*: cores integrated for research
*CV*: curriculum vitae
*DOPS*: direct observation of procedural skills
*EACCME*: European Council for Continuous Medical Education
*ESMSC*: Essential Skills in the Management of Surgical Cases
*EU*: European Union
*WTAS*: Westside Anxiety Score
*NASCE*: Network of Accredited Skills Centres in Europe
*ISCP*: Intercollegiate Surgical Curriculum Programme
